# Successful Re-Closure with Adjunctive Retention Sutures for Fascial Dehiscence after Mesh-Mediated Fascial Traction in Open Abdomen Management for Perforated Sigmoid Diverticulitis: A Case Report

**DOI:** 10.70352/scrj.cr.26-0573

**Published:** 2026-09-30

**Authors:** Tomohiro Takeda, Sato Nishida, Katsuro Enomoto, Daisuke Koro, Shoichiro Mizukami, Hiroyoshi Iwata, Masahide Otani, Mizuho Ohara, Chikayoshi Tani, Hiroyuki Takahashi, Tatsuya Shonaka, Koji Imai, Kimiharu Hasegawa, Hideki Yokoo

**Affiliations:** Division of Gastroenterological and Transplant Surgery, Department of Surgery, Asahikawa Medical University, Asahikawa, Hokkaido, Japan

**Keywords:** sigmoid diverticular perforation, septic shock, open abdomen, mesh-mediated fascial traction, abdominal wall dehiscence, adjunctive retention sutures, delayed fascial closure

## Abstract

**INTRODUCTION:**

Open abdomen management (OAM) is sometimes required in patients with severe peritonitis and septic shock when primary fascial closure is unsafe or impossible. Mesh-mediated fascial traction (MMFT) combined with negative pressure wound therapy (NPWT) is a useful strategy for delayed fascial closure. However, fascial dehiscence may still occur after definitive closure in patients with multiple risk factors for impaired wound healing. Reports describing retention sutures as a salvage adjunct for fascial dehiscence after MMFT are scarce. We report a high-risk case in which re-closure with adjunctive retention sutures maintained abdominal wall closure after fascial dehiscence following staged closure.

**CASE PRESENTATION:**

A man in his 60s with obesity and ongoing corticosteroid therapy for interstitial pneumonia was transferred to our hospital with upper abdominal pain and profound septic shock. CT showed extraluminal gas predominantly in the upper abdomen and pelvic ascites, but the perforation site was unclear. Emergency exploratory laparotomy revealed perforated sigmoid diverticulitis. Because of severe hemodynamic instability, segmental sigmoid colectomy was performed, and OAM was initiated. After second-look surgery, primary fascial closure remained difficult because of marked bowel edema and obesity. MMFT combined with NPWT was therefore performed. The abdominal wall defect gradually decreased, and fascial closure was achieved on POD 13. However, fascial dehiscence occurred on POD 17, with the small intestine in direct contact with the fascial sutures. On POD 18, re-closure was performed using fascial re-suturing combined with 4 retention sutures. During closure, the fascia was gradually approximated toward the midline with manual lateral-to-medial compression of the trunk, and the skin was left open with continued NPWT. The patient was transferred for rehabilitation on POD 100. At 1-year follow-up, the wound was fully epithelialized, and no obvious incisional hernia was observed.

**CONCLUSIONS:**

Fascial dehiscence may occur even after staged closure with MMFT and NPWT in high-risk patients. Adjunctive retention sutures may be a salvage option for re-closure when conventional fascial re-suturing alone is considered insufficient.

## Abbreviations

ABRA
: abdominal re-approximation anchor
ACS
: abdominal compartment syndrome
MMFT
: mesh-mediated fascial traction
NPWT
: negative pressure wound therapy
OAM
: open abdominal management

## INTRODUCTION

OAM is sometimes required in patients with severe peritonitis, septic shock, or marked visceral edema when primary fascial closure is unsafe or impossible. However, prolonged OAM may lead to lateral fascial retraction, difficulty in delayed fascial closure, and planned ventral hernia. Therefore, achieving delayed primary fascial closure while minimizing abdominal wall tension remains an important goal in critically ill surgical patients.

MMFT combined with NPWT has been reported as an effective dynamic fascial traction strategy for OAM.^1,2)^ Nevertheless, fascial dehiscence may still occur after definitive closure, particularly in patients with risk factors for impaired wound healing.^3)^

Retention sutures have historically been used to reduce tension on abdominal wall closure, but their role remains controversial because of possible postoperative pain, local suture-related complications, and increased intra-abdominal pressure.^4–6)^ Although retention sutures have been evaluated as a prophylactic adjunct in high-risk laparotomy patients, their use as a salvage adjunct for fascial dehiscence after staged closure with MMFT and NPWT remains unclear.

Herein, we report a case of perforated sigmoid diverticulitis with septic shock requiring OAM, in which re-closure with adjunctive retention sutures was successfully performed after fascial dehiscence following staged closure with MMFT and NPWT.

## CASE PRESENTATION

A man in his 60s with a BMI of 30.35 kg/m^2^ was transferred to our hospital with suspected gastrointestinal perforation. He had a history of open cholecystectomy and selective vagotomy through upper and lower midline incisions and a right upper paramedian incision. One month before presentation, he had received steroid pulse therapy for interstitial pneumonia and was taking prednisolone 40 mg/day at onset.

He presented with upper abdominal pain. On arrival, he was in profound shock: heart rate, 169 beats/min; respiratory rate, 34 breaths/min; blood pressure, 71/47 mmHg; and oxygen saturation, 95% with oxygen at 2 L/min. Blood gas analysis showed severe lactic acidosis, with a lactate level of 21 mmol/L. CT showed extraluminal gas predominantly in the upper abdomen and pelvic ascites, but the perforation site could not be identified (**Fig. 1A**, **1B**). Emergency exploratory laparotomy was performed for gastrointestinal perforation with septic shock.

**Fig. 1:**
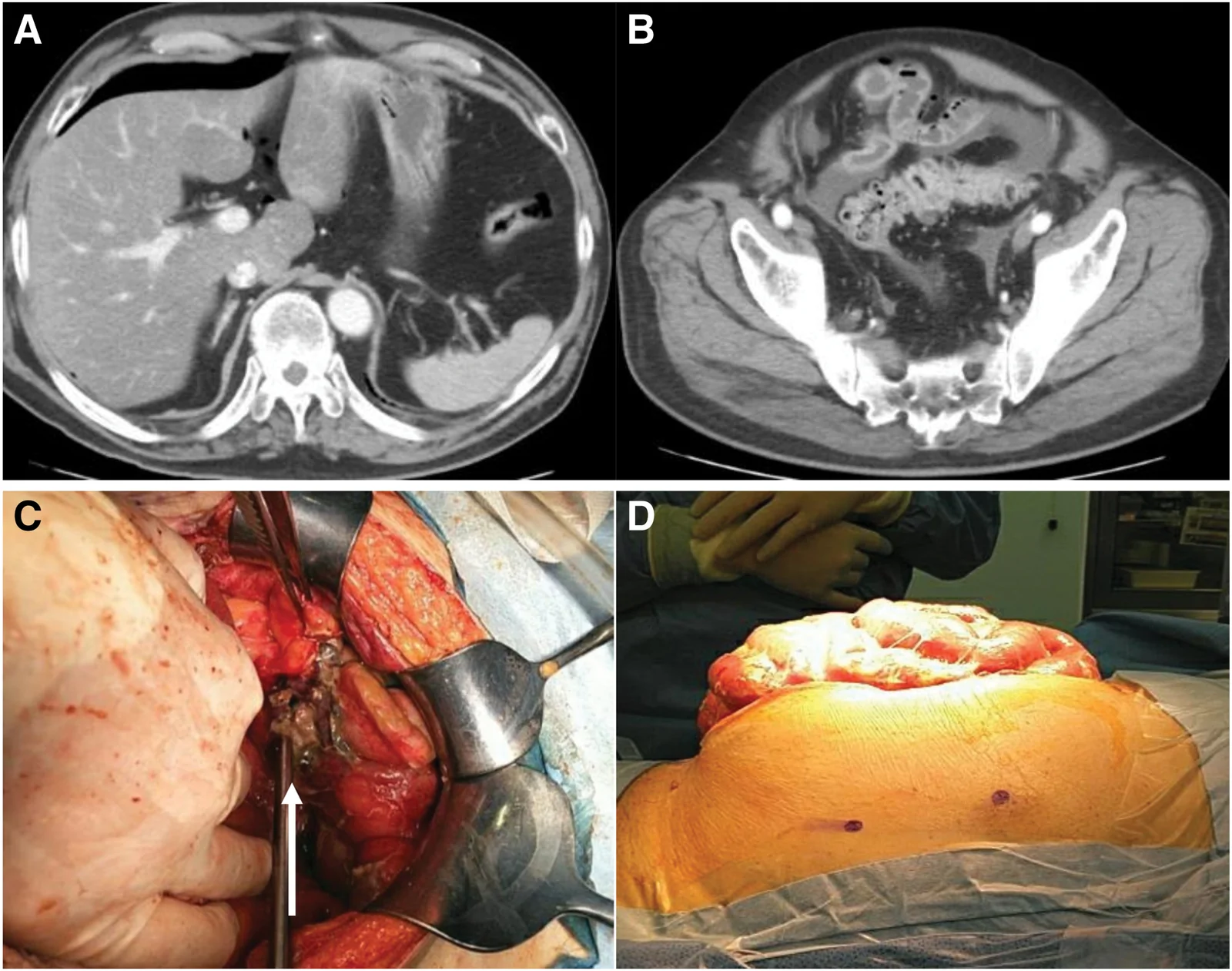
Initial CT and intraoperative findings. CT on arrival showed free air predominantly in the upper abdomen, whereas ascites was more prominent than free air in the lower abdomen (**A**, **B**). Intraoperative findings revealed perforation of a sigmoid diverticulum (white arrow) (**C**). During the second-look operation, abdominal closure was difficult because of marked bowel edema and obesity (**D**).

Intraoperatively, perforation of a sigmoid diverticulum was identified (**Fig. 1C**). Because of severe hemodynamic instability, only segmental resection of the sigmoid colon was performed, and OAM was initiated using the AbThera Open Abdomen Negative Pressure Therapy System (3M, St. Paul, MN, USA). Postoperatively, the patient required mechanical ventilation, high-dose vasopressors, polymyxin B-immobilized fiber column direct hemoperfusion for 48 h, and continuous hemodiafiltration using a polymethyl methacrylate membrane. On POD 3, second-look surgery was performed. Additional resection of the rectosigmoid colon, including the diverticular lesion, was performed; the AbThera system was exchanged; and OAM was continued because marked bowel edema made abdominal closure difficult (**Fig. 1D**).

On POD 6, surgery was performed with the intention of abdominal closure. However, the abdominal wall defect, defined as the distance between the bilateral rectus sheaths, measured 15 cm in transverse diameter and 28 cm in longitudinal diameter. Because of obesity and bowel edema, primary closure was considered difficult. A single-barrel descending colostomy was created, and MMFT with NPWT was performed. A 26 × 36-cm polypropylene mesh (Bard Mesh; Davol, Warwick, RI, USA) was used as the temporary traction mesh and fixed with continuous No. 1 polypropylene sutures (SURGIPRO; Covidien, Mansfield, MA, USA) incorporating the anterior rectus sheath, rectus muscle, and peritoneal layer to obtain secure fixation. The visceral protective layer of the AbThera system was placed beneath the peritoneum, and the bilateral mesh edges were approximated and rolled up toward the midline, followed by continuous suturing with 1-0 nylon. This procedure reduced the transverse diameter of the abdominal wall defect to 9 cm. NPWT using the AbThera system was then resumed, and the operation was completed after stoma creation (**Fig. 2**).

**Fig. 2:**
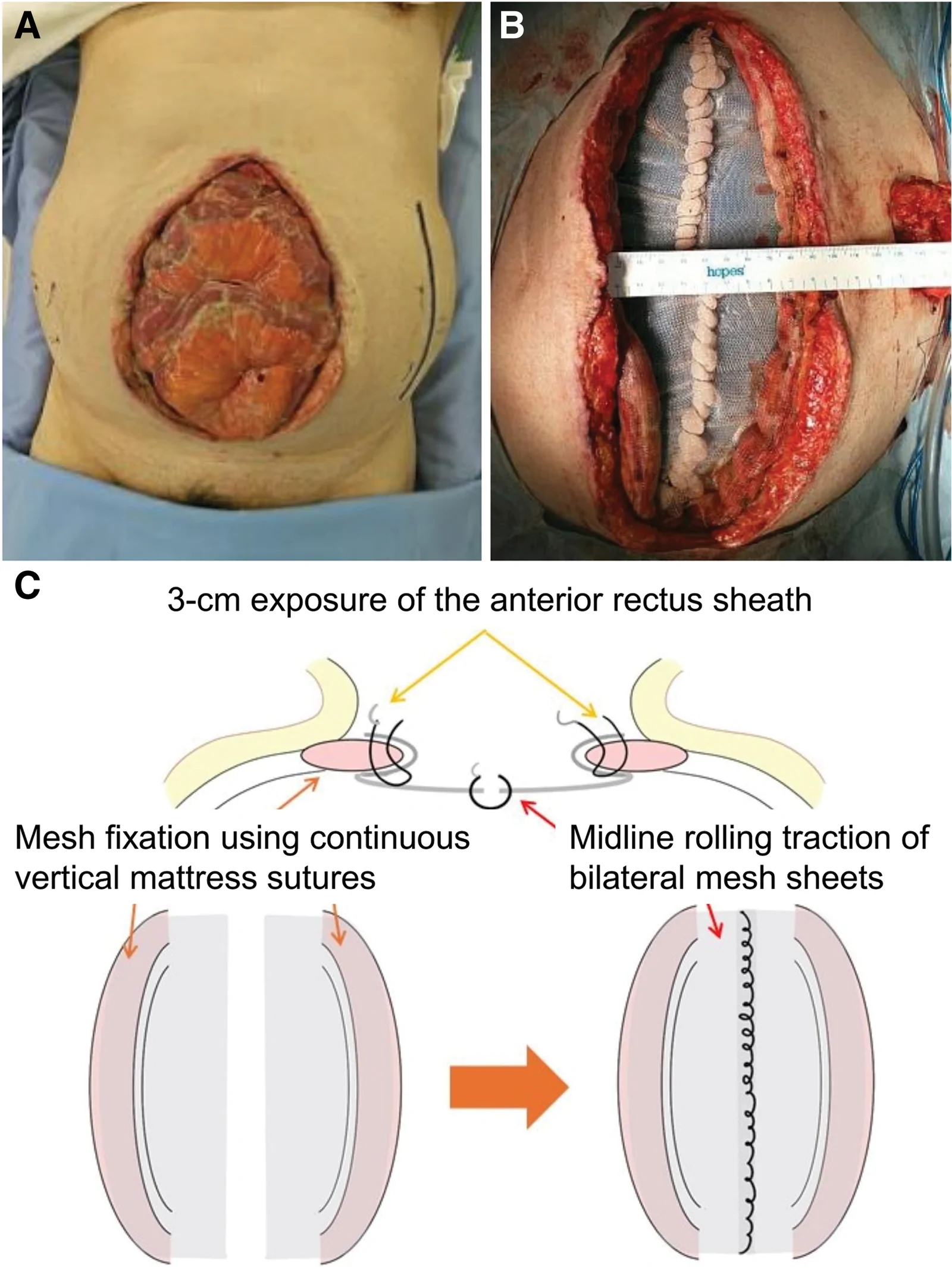
Initial MMFT. The first MMFT procedure was performed on POD 6. After removal of the AbThera system, the transverse diameter of the open abdominal wall was 15 cm (**A**), which was reduced to 9 cm after MMFT (**B**). A schematic illustration of the MMFT technique is shown in (**C**). MMFT, mesh-mediated fascial traction

On POD 8 and POD 10, MMFT with NPWT was continued, and tracheostomy was performed on POD 8 for anticipated prolonged respiratory management. At each MMFT procedure, the mesh itself was not replaced. The previous sutures between the bilateral mesh edges were removed, the abdominal cavity was irrigated, and the visceral protective layer of the AbThera system was exchanged after intra-abdominal lavage. The bilateral mesh edges were rolled medially toward the midline, temporarily held with hemostatic forceps, further rolled around the forceps to apply gradual traction, and then re-sutured in the midline with continuous 1-0 nylon sutures. This staged traction reduced the transverse fascial defect from 15 to 9 cm on POD 6, from 9 to 4 cm on POD 8, and from 4 to 3 cm on POD 10 (**Fig. 3A**, **3B**). During MMFT, intra-abdominal pressure was monitored in the ICU using bladder pressure measurements to detect ACS. On POD 13, the mesh and visceral protective layer of the AbThera system were removed, and fascial closure was achieved. There was almost no adhesion between the mesh and the intra-abdominal organs; when minor adhesions were present, they were easily separated. The mesh was removed without difficulty, and no bowel injury occurred. Adhesions between bowel loops were not forcibly dissected. No mesh infection was observed. However, a crush injury of the rectus sheath and rectus muscle was noted at the mesh fixation site. Fascial closure was achieved using continuous 0 Biosyn sutures (Covidien, Mansfield, MA, USA), followed by NPWT (**Fig. 3C**).

**Fig. 3:**
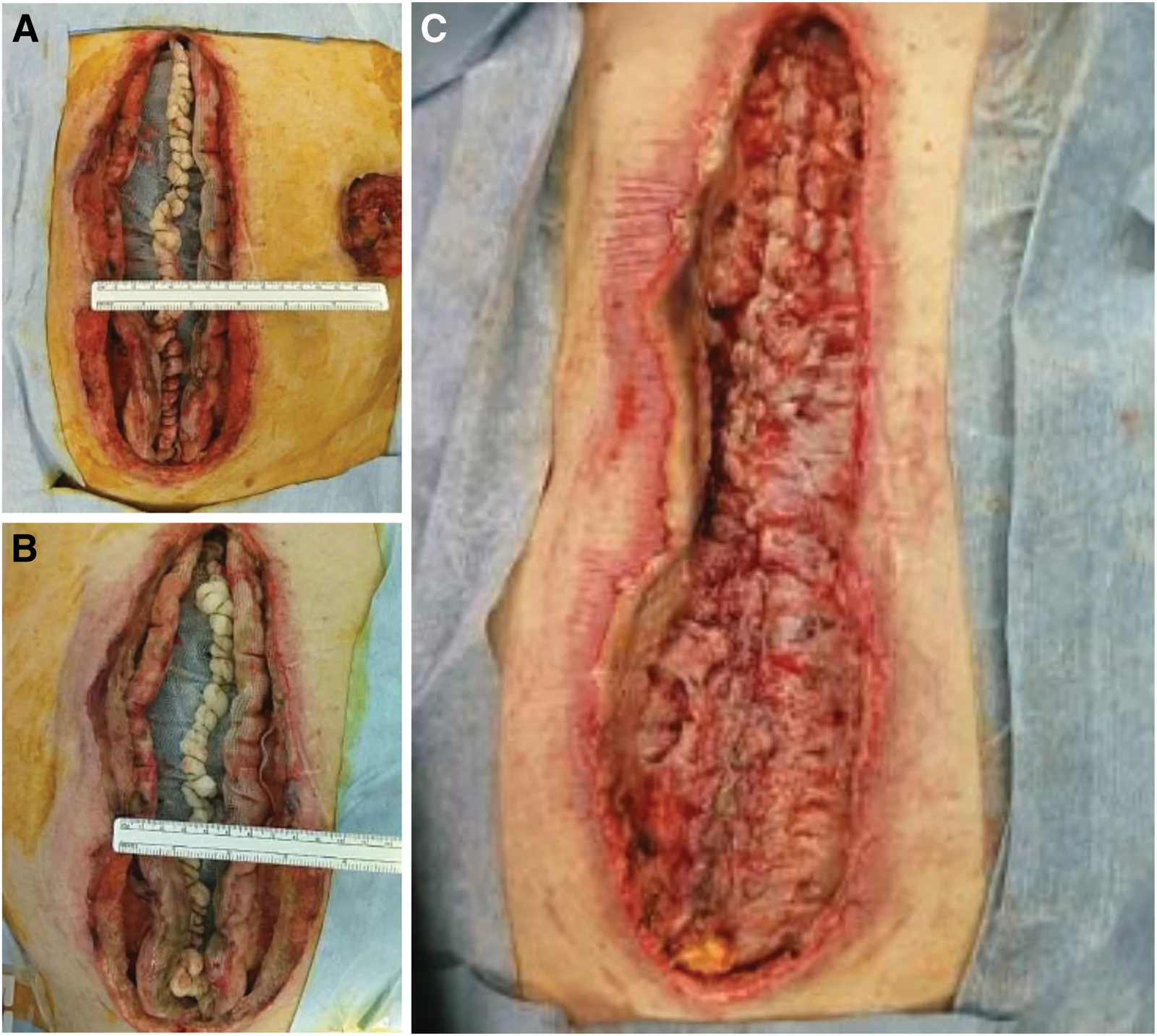
Clinical course until first abdominal closure. The transverse diameter of the open abdominal wall decreased to 4 cm on POD 8 (**A**) and to 3 cm on POD 10 (**B**). The first abdominal closure was performed on POD 13 (**C**).

On POD 17, the patient was discharged from the ICU after an 18-day stay. Bladder pressure was monitored in the ICU from POD 1 to POD 17 to detect intra-abdominal hypertension or ACS. Immediately after the initial fascial closure following MMFT on POD 13, bladder pressure transiently increased to 17 mmHg. Except for this single measurement, bladder pressure remained at or below 15 mmHg during the monitoring period. No clinical findings suggestive of ACS were observed.

After the initial fascial closure on POD 13, NPWT was continued. On POD 17, fascial dehiscence with bowel exposure was identified, and NPWT was discontinued. At that time, the small intestine was exposed beneath the disrupted fascial closure and was in direct contact with the fascial sutures. The fascial sutures were not broken but had shifted to one side. The abdominal wall tissue around the suture line appeared severely crushed and fragile, suggesting that the weakened tissue could not tolerate the closure tension and had torn. Although infection or tissue necrosis could not be completely excluded, no obvious purulent drainage was observed.

On POD 18, re-closure with adjunctive retention sutures was performed. The previous fascial sutures were removed, and crushed tissue was debrided as much as possible. Four retention sutures using 1-0 nylon were placed evenly along the wound, approximately 3 cm from the wound edge. Each suture was passed from the skin to the posterior rectus sheath while avoiding incorporation of the peritoneum. Fascial re-suturing was then performed using 1-0 nylon vertical mattress sutures (CROWNJUN Nylon Suture; Kono Seisakusho, Ichikawa, Japan), while manual lateral-to-medial compression of the trunk was applied to gradually approximate the fascia toward the midline. The retention sutures were passed through Nelaton tubes (Terumo Corporation, Tokyo, Japan) to prevent direct contact between the nylon sutures and the skin. Aquacel Ag (Convatec, Deeside, UK) was additionally inserted at sites where the suture could come into direct contact with the skin. The retention sutures were tied while leaving approximately 2 fingerbreadths of space beneath the sutures to avoid excessive tightening, and the skin was left open (**Fig. 4**).

**Fig. 4:**
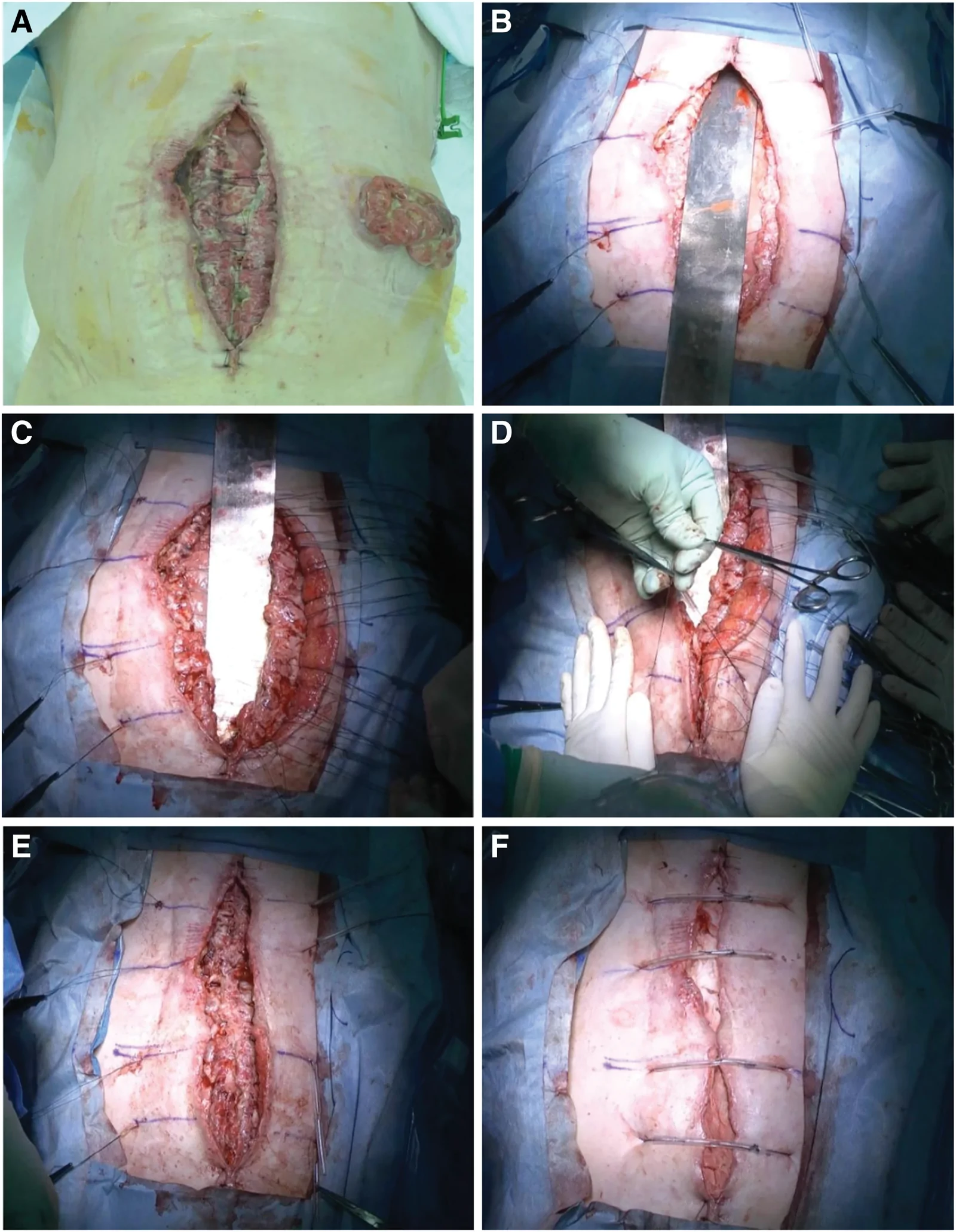
Re-closure with adjunctive retention sutures. Re-closure with adjunctive retention sutures was performed for fascial dehiscence on POD 18. The fascial sutures were in direct contact with the small intestine due to fascial dehiscence (**A**). Widely placed retention sutures using 1-0 nylon were applied (**B**). After securing the rectus sheath, vertical mattress sutures using 1-0 nylon were placed (**C**). During fascial suturing, traction on the retention sutures and manual tension reduction were applied simultaneously (**D**), and the fascia was re-sutured (**E**). After tying the retention sutures, the skin was left open (**F**).

After re-closure, serial debridement and wound care using a Sorbact dressing (Abigo Medical, Mölndal, Sweden) were continued. NPWT was restarted on POD 43 and discontinued on POD 53. The retention sutures were removed on POD 50. Fiblast Spray (Kaken Pharmaceutical, Tokyo, Japan) was also used to promote wound healing. A deep skin ulcer and associated pain developed at part of the retention suture site, but these improved after removal of the retention sutures. No abscess formation was observed. On POD 100, the patient was transferred to the referring hospital for rehabilitation. At the 1-year follow-up, the wound was fully epithelialized, and no obvious incisional hernia was detected by physical examination and inspection of the abdominal wall in the frontal and lateral positions. CT was not performed at that time (**Fig. 5**).

**Fig. 5:**
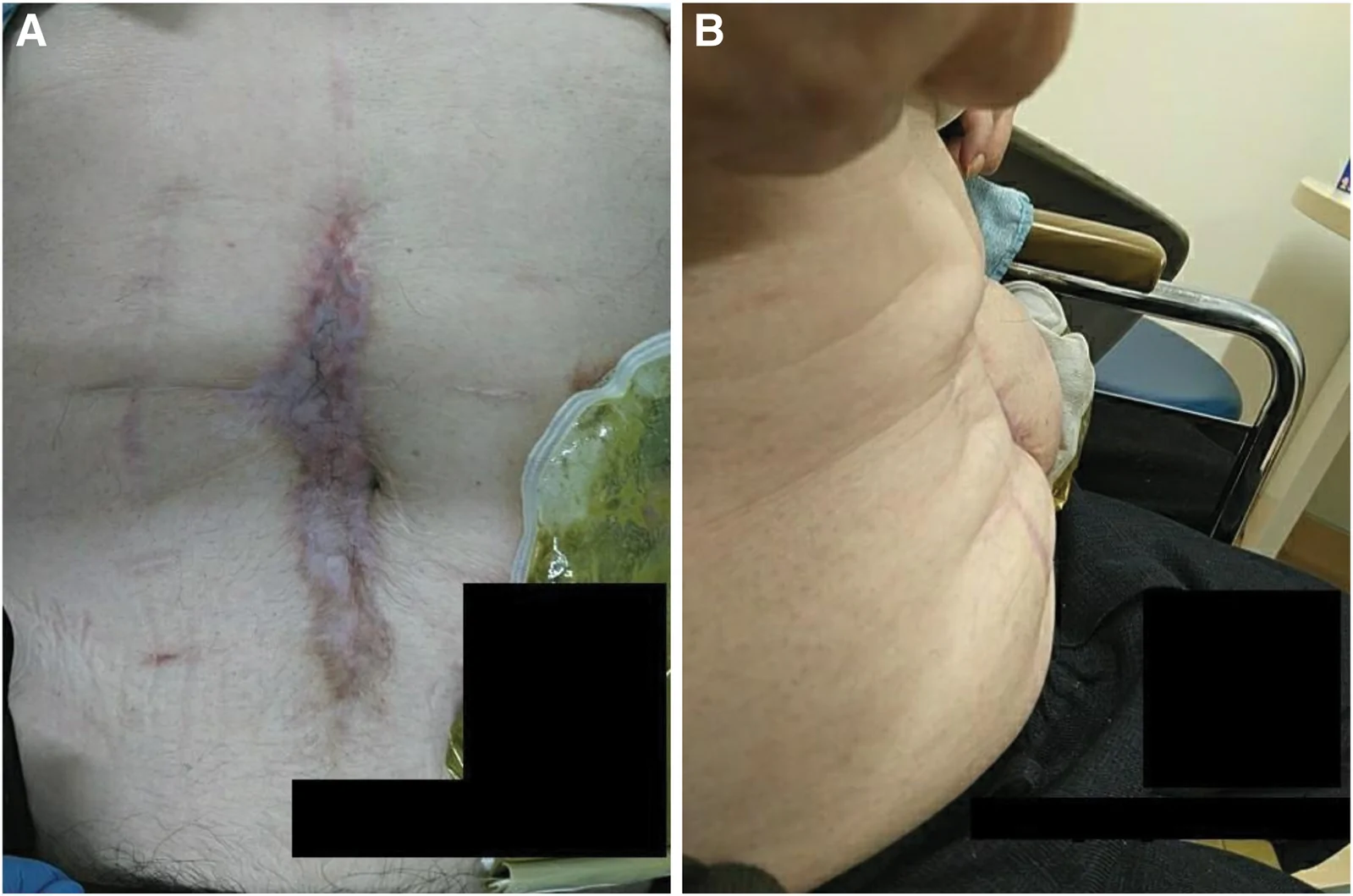
One-year postoperative follow-up. At the 1-year postoperative follow-up, the wound was fully epithelialized, and no obvious incisional hernia was observed in the frontal view (**A**) and lateral view (**B**).

## DISCUSSION

The significance of the present case is that abdominal wall closure was maintained by re-closure with adjunctive retention sutures in a high-risk patient who developed fascial dehiscence despite staged fascial closure with MMFT and NPWT. MMFT combined with NPWT has been described as a dynamic fascial traction technique for delayed fascial closure after OAM.^7–9)^ In addition to MMFT, several other dynamic fascial traction or staged closure techniques, including dynamic or continuous retention sutures, the ABRA system, the Wittmann patch, and intraoperative fascial traction, have been reported to reduce fascial lateralization and facilitate fascial closure.^3,10–12)^ However, these techniques are primarily used during OAM or before definitive closure to approximate the fascia toward the midline. In contrast, the retention sutures used in the present case were applied after fascial dehiscence had occurred following definitive closure with MMFT, as a salvage adjunct to distribute tension during re-closure. Although tension reduction sutures have been used to support definitive closure in prolonged open abdomen,^13)^ to our knowledge, reports describing conventional retention sutures as a salvage adjunct for fascial dehiscence after definitive closure with MMFT remain scarce. Therefore, this case may provide useful insight into re-closure strategies for severe abdominal wall closure difficulty.

The use of retention sutures remains controversial. Khorgami et al.^5)^ reported that prophylactic retention sutures reduced postoperative wound dehiscence in high-risk patients undergoing midline laparotomy who had multiple risk factors for impaired wound healing. In contrast, a prospective randomized study by Rink et al.^6)^ showed that retention sutures increased postoperative pain and were associated with local complications and early removal due to pain. Tang et al.^14)^ also reported that retention sutures may reduce wound dehiscence but may increase bladder pressure and postoperative pain. Therefore, routine prophylactic use of retention sutures for abdominal wall closure should be considered with caution, and careful patient selection and avoidance of excessive tightening are important, particularly in patients at risk of increased intra-abdominal pressure.

In the present case, retention sutures were not used as a routine adjunct at the first abdominal closure. Instead, they were used as a salvage option at the time of re-closure because fascial dehiscence occurred after staged closure with MMFT and NPWT. The patient had multiple risk factors for fascial dehiscence, including obesity, severe sepsis, ongoing corticosteroid therapy, repeated laparotomies, bowel edema after OAM, and a fragile rectus sheath. Risk factors for abdominal wound dehiscence, such as obesity, chronic pulmonary disease, corticosteroid use, sepsis, emergency surgery, and wound infection, have been reported, and the importance of identifying high-risk patients during the perioperative period has been emphasized.^4)^ Therefore, even after staged fascial closure with MMFT and NPWT, the risk of recurrent dehiscence was considered to persist in the present case.

In contaminated abdominal fields, permanent mesh implantation for abdominal wall reconstruction should be approached cautiously because of the risk of mesh infection. However, the mesh used in MMFT is a temporary traction device rather than a permanent prosthesis. In the present case, MMFT was initiated on POD 6, after source control had been achieved and the hemodynamic status had improved, but before further lateral fascial retraction progressed. The visceral protective layer of the AbThera system was interposed to avoid direct contact between the mesh and intra-abdominal organs. We considered that excessively early mesh placement might increase infection risk, whereas excessive delay might worsen lateral fascial retraction and make delayed primary closure more difficult. Thus, temporary mesh placement for MMFT may be considered once source control is achieved and staged fascial traction becomes necessary.

At the initial fascial closure, continuous 0 Biosyn sutures were used to distribute tension evenly and avoid prolonging operative time in a critically ill patient. Because dehiscence occurred very early after closure, absorption of the suture material itself was unlikely to have been the direct cause. However, in retrospect, continuous closure with an absorbable suture may have provided insufficient support in this high-risk setting, in which tissue fragility and local tissue injury associated with MMFT were present. Continuous suturing may distribute tension along the closure line, but failure at one point can compromise the entire closure, whereas interrupted sutures may facilitate repair if fascial tearing occurs. Therefore, the choice of suture material and closure technique after MMFT should be carefully considered in patients at high risk for fascial dehiscence.

The intraoperative findings at re-closure also suggested mechanical failure of weakened abdominal wall tissue. The fascial sutures had not broken but had shifted to one side, and the abdominal wall tissue around the suture line appeared severely crushed and fragile. These findings suggested that the weakened fascia and abdominal wall tissue failed because of tearing or suture pull-through under closure tension, rather than suture breakage. Although infection or tissue necrosis could not be completely excluded, no obvious purulent drainage was observed.

Local tissue injury related to MMFT may also have contributed to fascial dehiscence. Although no adhesion-related bowel injury or mesh infection occurred at mesh removal, crush injury of the rectus sheath and rectus muscle was observed at the mesh fixation site. During the initial MMFT procedure, the mesh was fixed to relatively deep layers, including the rectus muscle, to improve fixation strength. However, standard MMFT techniques emphasize taking only the fascia in the stitches and avoiding muscle penetration to prevent muscle ischemia. Thus, incorporation of the rectus muscle may increase fixation strength but may also increase the risk of muscle ischemia or crush injury during traction. In the present case, such tissue injury may have contributed to the subsequent fascial dehiscence, particularly in the setting of fragile abdominal wall tissue caused by severe sepsis, corticosteroid therapy, obesity, and repeated surgeries. Gentle mesh fixation without excessive exposure or dissection of the anterior rectus sheath, and without incorporating the rectus muscle, may be important to reduce local tissue injury during MMFT in high-risk patients.

At re-closure, the retention sutures were not simply tightened forcefully. Instead, the fascia was gradually approximated toward the midline while manual lateral-to-medial compression of the trunk was applied, and vertical mattress sutures were placed to securely incorporate the fragile rectus sheath. The skin was not closed primarily and was left open, which may have helped avoid excessive tension on the skin and subcutaneous tissue. Intra-abdominal pressure monitoring was also important during staged fascial approximation and closure because excessive negative pressure or fascial traction may increase the risk of intra-abdominal hypertension or ACS. In the present case, bladder pressure transiently increased to 17 mmHg immediately after the first fascial closure, but otherwise remained at or below 15 mmHg. Although the maximum value corresponded to grade II intra-abdominal hypertension, it remained below 20 mmHg and was not associated with clinical findings suggestive of ACS.^15)^ If a clinically significant increase in bladder pressure or organ dysfunction suggestive of ACS had occurred, we would have considered reducing the negative pressure setting of NPWT, loosening MMFT traction, or releasing the fascial traction or closure if necessary, followed by continued temporary abdominal closure with NPWT until intra-abdominal pressure and organ function improved.

Component separation techniques were also considered to reduce tension during fascial closure. Adjunctive left-sided unilateral transversus abdominis release was considered; however, right-sided release was avoided because of a previous right paramedian incision, and additional dissection was considered potentially hazardous in the setting of severe sepsis, obesity, corticosteroid therapy, repeated laparotomies, and possible local tissue injury associated with MMFT. Anterior component separation was also considered because it can provide substantial medial advancement of the abdominal wall. However, it was not selected because of the need for wide subcutaneous dissection and the associated risk of skin flap ischemia, skin necrosis, and wound complications.^16)^ Given the fragile abdominal wall tissue, additional dissection was considered potentially harmful. In addition, we considered preserving component separation as a future reconstructive option if an incisional hernia developed. Therefore, staged fascial traction with MMFT and NPWT was prioritized, followed by salvage re-closure with adjunctive retention sutures after fascial dehiscence.

This report describes a single case, and the efficacy of retention sutures cannot be generalized. In addition, the maintenance of re-closure after fascial dehiscence may not have been attributable to retention sutures alone, but rather to multidisciplinary management, including infection control, nutritional support, corticosteroid tapering, wound management with NPWT, and rehabilitation. Therefore, we cannot conclude that retention sutures should be routinely used for fascial dehiscence after MMFT. Nevertheless, in selected patients with multiple risk factors for impaired wound healing in whom conventional fascial re-suturing alone is considered insufficient, re-closure with adjunctive retention sutures may be a salvage option to maintain abdominal wall closure. Another limitation is that CT was not performed at the 1-year follow-up. Therefore, although no obvious incisional hernia was detected by physical examination, a small or subclinical incisional hernia could not be completely excluded.

## CONCLUSIONS

In selected high-risk patients, fascial dehiscence may occur even after staged closure with MMFT and NPWT. Re-closure with adjunctive retention sutures may be a useful salvage option to distribute abdominal wall tension and maintain fascial closure after dehiscence following MMFT. This strategy should not be considered a routine adjunct to MMFT, but it may provide an additional option for difficult abdominal wall re-closure when conventional fascial resuturing alone is considered insufficient.

## DECLARATIONS

### Use of artificial intelligence (AI) tools

The authors used ChatGPT (OpenAI, San Francisco, CA, USA) to assist with English language editing and readability improvement. No artificial intelligence tool was used for data collection, data analysis, image generation or modification, or clinical interpretation. The authors reviewed and edited all AI-assisted text and take full responsibility for the content of the manuscript.

### Funding

None.

### Authors’ contributions

TT drafted the manuscript, performed the surgery, and was responsible for the patient’s perioperative management as the attending surgeon.

MaO contributed to surgical planning and performed the retention suture procedure.

SN, KE, DK, SM, HI, MiO, CT, HT, TS, KI, and KH assisted with the surgeries and contributed to perioperative treatment and patient care.

HY supervised the study and is directly responsible for the manuscript.

All authors have reviewed and approved the final manuscript and agree to be accountable for all aspects of the work.

### Availability of data and materials

The datasets supporting the conclusions of this article are included within the article.

### Ethics approval and consent to participate

All procedures performed in the study were in accordance with the ethical standards of the institutional research committee and with the 1964 Declaration of Helsinki and its later amendments or comparable ethical standards. This article does not contain any animal studies.

### Consent for publication

Informed consent to publish has been obtained.

### Competing Interests

The authors declare that they have no conflict of interest to disclose.

## References

[1] Atema JJ, Gans SL, Boermeester MA. Systematic review and meta-analysis of the open abdomen and temporary abdominal closure techniques in non-trauma patients. World J Surg 2015; 39: 912–25. 10.1007/s00268-014-2883-6 25446477

[2] Mahoney EJ, Bugaev N, Appelbaum R, et al. Management of the open abdomen: a systematic review with meta-analysis and practice management guideline from the Eastern Association for the Surgery of Trauma. J Trauma Acute Care Surg 2022; 93: e110–8. 10.1097/TA.0000000000003683 35546420

[3] Gäddnäs F, Saarnio J, Ala-Kokko T, et al. Continuous retention suture for the management of open abdomen: a high rate of delayed fascial closure. Scand J Surg 2007; 96: 301–7. 10.1177/145749690709600408 18265858

[4] van Ramshorst GH, Nieuwenhuizen J, Hop WC, et al. Abdominal wound dehiscence in adults: development and validation of a risk model. World J Surg 2010; 34: 20–7. 10.1007/s00268-009-0277-y PMC2795859 19898894

[5] Khorgami Z, Shoar S, Laghaie B, et al. Prophylactic retention sutures in midline laparotomy in high-risk patients for wound dehiscence: a randomized controlled trial. J Surg Res 2013; 180: 238–43. 10.1016/j.jss.2012.05.012 22677612

[6] Rink AD, Goldschmidt D, Dietrich J, et al. Negative side-effects of retention sutures for abdominal wound closure. A prospective randomised study. Eur J Surg 2000; 166: 932–7. 10.1080/110241500447083 11152253

[7] Petersson U, Acosta S, Björck M. Vacuum-assisted wound closure and mesh-mediated fascial traction—a novel technique for late closure of the open abdomen. World J Surg 2007; 31: 2133–7. 10.1007/s00268-007-9222-0 17879112

[8] Acosta S, Bjarnason T, Petersson U, et al. Multicentre prospective study of fascial closure rate after open abdomen with vacuum and mesh-mediated fascial traction. Br J Surg 2011; 98: 735–43. 10.1002/bjs.7383 21462176

[9] Morris PD, Allaway MGR, Wright D. How to do mesh-mediated fascial traction for delayed primary closure of the open abdomen. ANZ J Surg 2023; 93: 1999–2002. 10.1111/ans.18474 37128158

[10] Iype S, Butler A, Jamieson N, et al. Delayed dynamic abdominal wall closure following multi-visceral transplantation. Int J Surg Case Rep 2014; 5: 988–91. 10.1016/j.ijscr.2014.08.006 PMC4276090 25460454

[11] Dennis A, Vizinas TA, Joseph K, et al. Not so fast to skin graft: transabdominal wall traction closes most “domain loss” abdomens in the acute setting. J Trauma Acute Care Surg 2013; 74: 1486–92. 10.1097/TA.0b013e3182924950 23694876

[12] Niebuhr H, Wegner F, Dag H, et al. Preoperative botolinum toxin A (BTA) and intraoperative fascial traction (IFT) in the management of complex abdominal wall hernias. Hernia 2024; 28: 2273–83. 10.1007/s10029-024-03156-z PMC11530493 39269518

[13] Yamataka T, Matsumoto S, Shimizu M. Delayed fascial closure for prolonged open abdomen. Trauma Surg Acute Care Open 2024; 9: e001524. 10.1136/tsaco-2024-001524 PMC11683964 39737146

[14] Tang H, Liu D, Qi HF, et al. Effect of retension sutures on abdominal pressure after abdominal surgery. Chin J Traumatol 2018; 21: 20–6. 10.1016/j.cjtee.2017.08.008 PMC6114125 29429775

[15] Kirkpatrick AW, Roberts DJ, De Waele J, et al. Intra-abdominal hypertension and the abdominal compartment syndrome: updated consensus definitions and clinical practice guidelines from the World Society of the Abdominal Compartment Syndrome. Intensive Care Med 2013; 39: 1190–206. 10.1007/s00134-013-2906-z PMC3680657 23673399

[16] Krpata DM, Blatnik JA, Novitsky YW, et al. Posterior and open anterior components separations: a comparative analysis. Am J Surg 2012; 203: 318–22. 10.1016/j.amjsurg.2011.10.009 22244073

